# Epidemiological Surveillance of Genetically Determined Microcephaly in Latin America: A Narrative Review

**DOI:** 10.3390/epidemiologia6030037

**Published:** 2025-07-14

**Authors:** Melissa Daniella Gonzalez-Fernandez, Karina Jiménez-Gil, Linda Garcés-Ramírez, Alejandro Martínez-Juárez, Elsa Romelia Moreno-Verduzco, Juan Mario Solís-Paredes, Javier Pérez-Durán, Johnatan Torres-Torres, Irma Eloisa Monroy-Muñoz

**Affiliations:** 1 Laboratory of Behavioral Physiology, Department of Physiology, National School of Biological Sciences, National Polytechnic Institute, Mexico City P.C. 07700, Mexico; mgonzalezf1001@alumno.ipn.mx (M.D.G.-F.); lgarces@ipn.mx (L.G.-R.); 2 Department of Reproductive and Perinatal Health Research, National Institute of Perinatology Isidro Espinosa de los Reyes, Mexico City P.C. 11000, Mexico; kjimenezg2100@alumno.ipn.mx (K.J.-G.); elsamover@yahoo.com (E.R.M.-V.); juan.solis@inper.gob.mx (J.M.S.-P.); jperezd@ipn.mx (J.P.-D.); johnatan.torres@inper.gob.mx (J.T.-T.); 3 Cellular Physiology Department, National Institute of Perinatology, Mexico City P.C. 11000, Mexico; alejandro.martinez@inper.gob.mx

**Keywords:** microcephaly, reduced head-circumference, genetic variants, chromosomal anomalies, monogenic syndromes, surveillance system, Latin America, Ibero America, ECLAMC, RyVECLAM, ReLAMC

## Abstract

**Background/Objectives:** Congenital microcephaly is a clinical manifestation with a heterogeneous etiology, and its epidemiological surveillance relies on the systematic identification of cases and investigation of their underlying causes to inform preventive strategies and improve prognostic assessments. In Latin America, despite the existence of congenital anomaly reporting programs since 1967, the surveillance of microcephaly only gained substantial attention following the Zika virus (ZIKV) epidemic in 2015. Since then, efforts have predominantly concentrated on cases of infectious origin, often at the expense of recognizing endogenous etiologies, particularly those of genetic nature. This review aims to examine the role of genetic alterations in microcephaly pathogenesis and evaluates the limitations of current surveillance systems. **Methods:** A literature review centered on syndromic and non-syndromic genetic etiologies, alongside an analysis of Latin American surveillance frameworks (ECLAMC, RyVEMCE, ICBDSR, ReLAMC) was performed. **Results:** The findings reveal improved case detection and increased reported prevalence; however, the proportion of genetically attributed cases has remained stable. No systematic studies were found identifying the most common genetic causes; instead, genetic investigations were limited to isolated cases with a family history. **Conclusions:** While epidemiological surveillance systems in Latin America have advanced in the reporting of congenital microcephaly cases, substantial gaps remain in case ascertainment and etiological investigation, particularly concerning genetic contributions

## 1. Introduction

Microcephaly is a structural brain defect characterized by an occipitofrontal head circumference that is more than two standard deviations below the mean, or below the 3rd percentile for height, sex, and gestational age [1,2,3]. It is classified as severe when the head circumference is more than three standard deviations below the mean. Microcephaly can be further categorized as primary or congenital when it is evident prenatally or at birth, or as secondary when the head circumference appears normal at birth, but cranial growth fails to progress adequately during the first year of life [2].

Microcephaly is considered a clinical sign, as it often reflects an underlying reduction in brain volume, which may result in abnormal neurodevelopment [4]. Any condition that disrupts fetal brain neurogenesis—including failures in neuronal proliferation, differentiation, or migration, as well as disruptions in DNA repair or apoptosis during pre- and postnatal neuronal development—can lead to microcephaly [1,2,4]. These causes are diverse and may include numerical or structural chromosomal abnormalities, single-gene mutations, intrauterine infections (such as TORCH or Zika virus), exposure to teratogenic substances, maternal metabolic disorders, severe malnutrition, or disruptive events like vascular accidents [1,3,4].

The global incidence of microcephaly varies widely depending on the population and diagnostic criteria, ranging from 1.3 to 150 per 100,000 births [2]. In the United States, the reported incidence between 2009 and 2013 was 8.7 cases per 10,000 births (0.87 per 1000), while in Portugal and the United Kingdom, rates ranged from 0.41 to 4.25 per 10,000 births [4]. For congenital microcephaly, incidence in the United States and Europe ranges between 0.58 and 1.87 per 10,000 births. In Latin America, data from the Latin American Collaborative Study of Congenital Malformations (ECLAMC) and the Latin American Network for Congenital Malformation Surveillance (ReLAMC) indicate that, up to 2014, the incidence of microcephaly ranged from 2.32 to 3.7 cases per 10,000 births. During the 2015 Zika virus (ZIKV) outbreak in Brazil, the incidence of microcephaly increased significantly, reaching 2.8 to 5.91 cases per 10,000 births in states with confirmed ZIKV transmission, compared to 0.6 cases per 10,000 births in regions without reported ZIKV activity [1,5,6].

While most of the literature attributes approximately one-third of microcephaly cases to genetic causes [1,2], a 2012 retrospective study in Argentina found that 69.5% of evaluated cases were associated with genetic factors [4]. This discrepancy raises important questions regarding the accuracy and consistency of microcephaly diagnosis, follow-up, and etiological classification across different countries.

Therefore, this study aims to review the genetic etiology of microcephaly, assess the extent to which such causes are reported in Latin America, and examine the strategies used for identifying and recording genetically linked cases. In particular, it seeks to determine whether it is possible to obtain clear, unconfounded data, especially in contexts where infectious causes—such as during the 2015 ZIKV outbreak—may overshadow or obscure genetic contributions.

## 2. Relevant Sections

### 2.1. Genetic Etiology of Congenital Microcephaly

Congenital microcephaly has a known cause in approximately 59% of cases. Among these, a genetic origin accounts for 30–45% [1,2,7], involving both structural and numerical chromosomal abnormalities as well as monogenic mutations. These genetic alterations may lead to microcephaly either in isolation or alongside other malformations. This section highlights only a subset of these genetic conditions, as the OMIM (Online Mendelian Inheritance in Man) database lists over 1100 entries associated with microcephaly. Table 1 provides an overview of syndromes involving major chromosomal alterations or monogenic mutations known to cause microcephaly [1].

### 2.2. Numerical and Structural Chromosomal Abnormalities Associated with Microcephaly

Numerical and structural chromosomal abnormalities, particularly trisomies and monosomies, are among the most prevalent genetic contributors to congenital microcephaly. Several well-characterized chromosomal syndromes include microcephaly as a recurrent phenotypic feature.

Trisomy 13 (Patau Syndrome) is a rare autosomal trisomy with an incidence of approximately 1 in 10,000 to 20,000 live births and a reported antenatal mortality rate of 95%. Microcephaly is present in approximately 86% of cases involving full trisomy 13—characterized by the presence of three complete copies of chromosome 13 due to meiotic nondisjunction, typically during meiosis II. In contrast, microcephaly is observed in 52% of mosaic trisomy 13 cases—where both normal and trisomic cell lines coexist—and in 29% of cases with partial trisomy 13q, which typically result from unbalanced translocations or inversions [8]. Phenotypically, Patau Syndrome is associated with profound midline defects, including holoprosencephaly (HPE), which arises from abnormal prechordal mesoderm fusion and is frequently accompanied by facial asymmetry [8,9,28].

Trisomy 21 (Down Syndrome) represents the most prevalent chromosomal aneuploidy, with a birth prevalence ranging from 1 in 319 to 1000 live births [9]. Neuropathological features include volumetric reduction in brain structures such as the hippocampus and cerebellum, often leading to microcephaly. Additionally, brachycephaly—characterized by an abnormally increased transverse cranial diameter—is commonly observed. These craniofacial features, along with other characteristic dysmorphisms, exhibit nearly complete phenotypic penetrance [10,11].

Trisomy 18 (Edwards Syndrome) is the second most common autosomal trisomy after Down Syndrome, with a global prevalence estimated at 1 in 3600 to 10,000 live births. Microcephaly is observed in 94% of cases with full trisomy 18, in 5% of mosaic cases, and in approximately 2% of partial trisomy cases. Clinical manifestations frequently include microphthalmia, hypertelorism, microretrognathia, and a high incidence (≥90%) of congenital skeletal and cardiac anomalies [12,13].

Among monosomies, Cri-du-Chat Syndrome (5p deletion syndrome) is the most frequently reported contiguous gene deletion disorder, with an incidence of 1 in 15,000 to 50,000 live births. This condition is characterized by a high prevalence of microcephaly (91.1%), distinctive craniofacial dysmorphisms, psychomotor and cognitive developmental delay, and a characteristic high-pitched cry reminiscent of a cat, from which the syndrome derives its name [14,29].

Wolf–Hirschhorn Syndrome, resulting from a terminal deletion of the short arm of chromosome 4 (4p16.3), has an estimated prevalence of 1 in 20,000 to 50,000 live births, with a female-to-male ratio of approximately 2:1. Microcephaly is present in approximately 90% of affected individuals. The syndrome is characterized by a distinctive craniofacial appearance often described as resembling a “Greek warrior helmet”, micrognathia, growth retardation, and a high incidence (93%) of seizure disorders [15,16,17]. Chromosomal abnormalities—particularly trisomies and monosomies—are among the most frequent genetic causes of congenital microcephaly. Several well-characterized syndromes include microcephaly as part of their clinical presentation.

### 2.3. Monogenic Syndromic Mutations Associated with Microcephaly

Monogenic syndromes represent a significant subset of genetic etiologies for congenital microcephaly. These conditions frequently involve mutations that affect critical neurodevelopmental pathways, often resulting in a constellation of systemic and craniofacial anomalies.

Smith–Lemli–Opitz Syndrome (SLOS) is a multiple congenital anomaly syndrome caused by a deficiency of the enzyme 7-dehydrocholesterol reductase, encoded by the *DHCR7* gene. This enzyme is essential in the biosynthesis of cholesterol, a key molecule in neuronal myelination and brain development. SLOS follows an autosomal recessive inheritance pattern and has an estimated incidence of 1 in 40,000 live births in the United States. Microcephaly is present in 80–84% of cases. Additional features include genital anomalies (70%), syndactyly of the toes, polydactyly of the hands, cleft lip and/or palate, intestinal dysmotility, and intellectual disability of variable severity [15,18,19].

Feingold Syndrome Type 1 results from pathogenic variants in the *MYCN* gene and follows autosomal dominant inheritance. To date, 69 families and 116 affected individuals have been reported. The phenotype includes microcephaly in approximately 86% of cases, digital anomalies (clinodactyly of the second and fifth digits, middle phalanx hypoplasia), dysmorphic facial features, tracheoesophageal fistula, duodenal atresia, and cognitive impairment. Notably, Feingold Syndrome Type 2, caused by mutations in the *MIR17HG* gene, also presents with microcephaly but lacks the gastrointestinal anomalies observed in Type 1 [15,20].

Cornelia de Lange Syndrome (CdLS) is a genetically heterogeneous disorder predominantly caused by De Novo heterozygous pathogenic variants in *NIPBL* (80% of cases), with less frequent involvement of *SMC1A* (5%), *HDAC8* (4%), *SMC3* (1–2%), *RAD21* (<1%), and *BRD4* (<1%). The inheritance is generally autosomal dominant, except for *SMC1A* and *HDAC8*, which are X-linked. The estimated prevalence is 1 in 50,000 live births in Europe [21]. Phenotypic features include distinct craniofacial dysmorphisms (arched eyebrows, synophrys), microcephaly (>90% of cases), growth retardation, limb reduction defects, oligodactyly, and varying degrees of intellectual disability [14,15,16].

Miller–Dieker Syndrome is a severe lissencephaly syndrome caused primarily by deletions in chromosome 17p13.3, encompassing the *PAFAH1B1* and *YWHAE* genes. These genes are critical regulators of neuronal migration. While most cases are genetic, some may be secondary to prenatal insults such as cytomegalovirus infection or placental insufficiency. The incidence is approximately 1 in 100,000 live births. Clinical manifestations include lissencephaly, congenital or postnatal-onset microcephaly, micrognathia, and multi-organ malformations affecting the heart, kidneys, and eyes [17,18,19].

Seckel Syndrome is a rare autosomal recessive disorder characterized by severe intrauterine and postnatal growth retardation, microcephaly, intellectual disability, and distinctive craniofacial features often described as “bird-headed.” The estimated incidence is 1 in 10,000 live births. Multiple causative genes have been identified, including *ATR*, *CENPJ*, *CEP152*, *CEP63*, *DNA2*, *NIN*, *NSMCE2*, *RBBP8*, and *TRAIP*, many of which are involved in DNA damage response and centrosome function [20,21,30].

### 2.4. Autosomal Dominant Primary Microcephaly

Autosomal dominant forms of primary microcephaly are less common. DYRK1A syndrome, caused by mutations in the *DYRK1A* gene, is a notable example. Microcephaly is present in approximately 95% of reported cases, along with other anomalies including dental defects, urogenital malformations, and lower limb abnormalities. To date, only 68 cases have been documented worldwide, including in adults. The syndrome demonstrates full phenotypic penetrance [22].

### 2.5. Autosomal Recessive Primary Microcephaly (MCPH)

Autosomal recessive primary microcephaly (MCPH) is characterized by isolated congenital microcephaly with severe intellectual disability, typically in the absence of other systemic or craniofacial abnormalities. Prevalence estimates range from 1 in 30,000 to 1 in 250,000 live births, varying by population [21,23,24].

Neurological deficits often include language delays, attention deficits, and in some cases, short stature, overlapping with Seckel Syndrome. Over 30 genes have been implicated in MCPH, with the most frequently mutated being *ASPM* (68.6%), *WDR62* (14.1%), and *MCPH1* (8%) [23].

**Figure 1:**
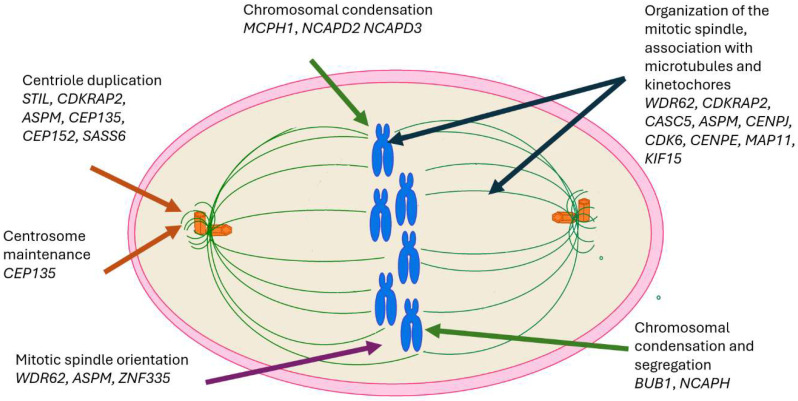
Genes involved in the formation of the mitotic spindle related with MCPH.

Most MCPH-related genes encode proteins that play key roles in centrosome function, mitotic spindle assembly, and chromosome segregation during neurogenesis. These include *MCPH1*, *WDR62*, *CDK5RAP2*, *CASC5*, *ASPM*, *CENPJ*, *STIL*, *CIT*, *CEP135*, *CEP152*, *CDK6*, *SASS6*, *KIF14*, *MAP11*, *NCAPD2*, *NCAPD3*, *NCAPH*, and *BUB1,* shown in Figure 1*.* Pathogenic variants disrupt neuronal progenitor cell division, leading to premature neurogenesis and a reduced neuronal population [24,25,26,27,31].

Some genes implicated in Seckel Syndrome also overlap with MCPH, notably *CENPJ*, *CDK5RAP2*, and *CEP152* [21,30].

In addition, several MCPH-associated genes are involved in DNA damage response pathways, such as *PHC1*, *MCPH1*, *CIT*, and *BUB1*, the latter being particularly sensitive to ionizing radiation. Other genes have broader cellular functions: *ZNF335* and *CENPE* encode transcriptional regulators. *MFSD2A* encodes a transporter for omega-3 fatty acids (notably DHA), crucial for blood–brain barrier function. *COPB2* is involved in non-clathrin vesicle trafficking from the Golgi to the endoplasmic reticulum. *NUP37* plays a role in nuclear pore complex assembly, facilitating macromolecular transport between nucleus and cytoplasm [24,32,33]. *ANKLE2* regulates nuclear envelope reassembly during mitosis. *LMNB1* and *LMNB2* encode nuclear lamina components; mutations can lead to myelin defects [34]. *RRP7A* is essential for ribosomal RNA processing, and mutations disrupt the cell cycle’s S phase [35]. *PDCD6IP* encodes a scaffolding protein involved in clathrin-mediated endocytosis and cytokinesis [36]. *WDFY3*, uniquely inherited in an autosomal dominant pattern, encodes ALFY, a key autophagy adaptor protein implicated in axonal development [21,24].

### 2.6. Microcephaly Associated with Impaired DNA Repair and Neuronal Migration

Beyond the classical genes associated with primary autosomal recessive microcephaly (MCPH), several additional genes involved in DNA damage response and neuronal migration are implicated in microcephaly, often within broader syndromic contexts.

Genes such as *NBN*, *RAD50*, *LIG4*, and *NHEJ1* are critical for maintaining genomic stability and repairing DNA double-strand breaks. *NBN* encodes nibrin, a protein essential for the cellular response to DNA damage and chromosomal integrity, particularly in the homologous recombination repair pathway. *RAD50* participates in DNA recombination, repair of double-strand breaks, and telomere maintenance. Both *LIG4* and *NHEJ1* are integral components of the non-homologous end joining (NHEJ) pathway, a major mechanism for repairing double-strand DNA breaks. Despite their autosomal recessive inheritance patterns, mutations in these genes are not typically classified under MCPH, as they are associated with additional syndromic manifestations, such as microcephalic primordial dwarfism [37].

Defects in neuronal migration represent another pathogenic mechanism leading to microcephaly, often accompanied by structural brain malformations such as lissencephaly, polymicrogyria, and cerebral hypoplasia. Several genes involved in cytoskeletal dynamics and neurodevelopmental signaling pathways have been implicated. *PAFAH1B1* (*LIS1*) encodes a subunit of the platelet-activating factor acetylhydrolase complex, which regulates dynein-mediated intracellular transport. Mutations result in impaired proliferation and migration of neuronal precursors from the ventricular to subventricular zones of the developing cortex. This gene follows an autosomal dominant inheritance pattern. *NDE1* is required for centrosome duplication and mitotic spindle formation. Loss-of-function variants lead to severe cortical malformations and are inherited in an autosomal recessive manner. *RELN* encodes reelin, a secreted extracellular matrix protein that guides neuronal positioning in the cerebral and cerebellar cortices. Mutations, typically autosomal recessive, result in disrupted cortical lamination. *ARX*, an X-linked transcription factor, is necessary for the maintenance and specification of neuronal subtypes in the cerebral cortex. Mutations cause a spectrum of neurodevelopmental disorders, often with microcephaly. The *TUBA1A*, *TUBB2B*, and *TUBB3* genes encode α- and β-tubulin subunits involved in microtubule formation. Mutations in these genes impair microtubule stability and neuronal migration and follow autosomal dominant inheritance [38,39].

### 2.7. Holoprosencephaly (HPE)

Holoprosencephaly (HPE) is a structural brain malformation resulting from incomplete division of the prosencephalon during the third to fourth week of embryogenesis. It is the most frequent malformation of the embryonic forebrain, with a birth prevalence estimated between 1 in 8000 and 1 in 16,000 live births, and as high as 1 in 250 conceptions due to high rates of prenatal lethality.

The clinical spectrum of HPE is highly variable and typically classified into three anatomical subtypes. Alobar HPE, the most severe form, characterized by a complete failure of forebrain division, resulting in a monoventricular brain and fused cerebral hemispheres. Semilobar HPE, marked by partial separation of the hemispheres, primarily affecting anterior structures. Lobar HPE, the mildest form, in which only the inferior frontal lobes fail to fully divide [40].

Craniofacial anomalies are frequently observed and may include hypertelorism, ocular colobomas, microtia, ethmocephaly, and in severe cases, cebocephaly or cyclopia.

Genetically, HPE is heterogeneous. The most commonly associated chromosomal anomalies are trisomy 13 and trisomy 18. However, monogenic forms have also been described, involving genes essential for midline development and Sonic Hedgehog (SHH) signaling. Pathogenic variants in *SHH*, *ZIC2*, *SIX3*, *TGIF1*, *PTCH1*, and *GLI2* have been reported. Notably, mutations in *GLI2* are typically associated with non-syndromic forms of HPE, though expressivity can vary widely even among carriers of the same mutation [38,39,41].

## 3. Epidemiological Surveillance of Congenital Microcephaly with Genetic Etiology in Latin America

Since 1967, the Latin American Collaborative Study of Congenital Malformations (ECLAMC) has operated across South America to identify congenital anomalies, elucidate their etiologies, and support primary prevention through research. Between 1997 and 2012, ECLAMC engaged a network of 261 voluntarily participating hospitals in Chile, Argentina, Bolivia, Brazil, Peru, Venezuela, and Colombia [42,43]. In Mexico, a parallel initiative—the Registry and Epidemiological Surveillance of External Congenital Malformations (RyVEMCE)—was launched in 1978 at the National Institute of Medical Sciences and Nutrition “Salvador Zubirán” and now encompasses 26 cities [44].

On a global scale, the International Clearinghouse for Birth Defects Surveillance and Research (ICBDSR) was established in 1974 and currently monitors over 4 million births annually, excluding Africa. ICBDSR collaborates with ECLAMC, RyVEMCE, and 26 hospitals in Nuevo León, Mexico [42,43,45,46].

Among ECLAMC’s notable contributions are the development of follow-up protocols and care guidelines for affected families, the creation of a photographic diagnostic guide, and the identification of associations between teratogenic exposures (e.g., industrial toxins) and congenital anomalies. Additionally, ECLAMC research highlighted the prevalence of a 5,10-methylenetetrahydrofolate reductase (MTHFR) variant in 10% of the studied population, necessitating folic acid supplementation—leading to the implementation of folate fortification in Chile in 2000 [42,43,45,47,48,49,50].

Since 2010, and prior to the Zika virus (ZIKV) epidemic, a diagnosis of microcephaly in Latin America was typically made when the newborn’s head circumference was more than three standard deviations (SD) below the mean. From 2016 onward, the diagnostic threshold was adjusted to two SD below the mean, based on hospital-specific growth charts or the Intergrowth-21st standards, in conjunction with additional clinical parameters assessed by the attending physician. These included prenatal diagnosis, known parental consanguinity (typically from second cousins or closer), documented prenatal exposure to teratogenic or infectious agents (such as those included in the TORCH syndrome), and maternal risk factors including metabolic conditions (e.g., diabetes mellitus), advanced maternal age, and place of residence.

In addition, microcephaly cases could be categorized by etiology. These classifications included associations with chromosomal (A1.1) or monogenic (A1.2) syndromes; environmental embryopathologies such as exposure to teratogens or congenital infections (A2); multiple microcephaly cases (B); microcephaly associated with central nervous system developmental anomalies (C); and isolated microcephaly (D). However, it is important to note that there have been persistent shortcomings in the follow-up and monitoring of reported cases, primarily due to limited resources in many of the hospital centers contributing data to the ECLAMC. Consequently, the true prevalence of microcephaly in the region may still be underestimated.

Following the Zika virus (ZIKV) epidemic in Brazil in 2015 and a subsequent increase in microcephaly cases across Latin America, the Latin American Network for Congenital Malformation Surveillance (ReLAMC) was established in 2016. ReLAMC, comprising 12 countries including Argentina, Brazil, Chile, Colombia, Costa Rica, Mexico, Nicaragua, Panama, Paraguay, and Uruguay, differs from ECLAMC in its approach: it analyzes population records rather than focusing on Case–Control surveillance. ReLAMC compared microcephaly incidence before (2010–2014) and during (2015–2017) the ZIKV epidemic and has since been integrated into the ICBDSR framework [46,51,52].

### 3.1. Prevalence of Congenital Microcephaly of Genetic Etiology in Latin America

Prior to 2015, the prevalence of microcephaly was likely underestimated by congenital malformation surveillance systems in Latin America. This underestimation was partly due to the restrictive diagnostic criterion at the time, which classified microcephaly only in newborns with head circumferences more than three standard deviations (SD) below the mean. Furthermore, case detection relied heavily on the initial diagnosis by the first-contact physician, typically a pediatrician, making the accuracy and completeness of reporting contingent on both the availability of resources at the healthcare facility and the physician’s expertise in identifying cases warranting further investigation. Before the ZIKV outbreak, the reported prevalence of microcephaly associated with chromosomal syndromes ranged from 0.18 to 0.55 per 10,000 births, while microcephaly due to monogenic syndromes was reported at a rate of 0.16 to 0.48 per 10,000 births. The surge in microcephaly cases during the ZIKV epidemic prompted increased attention to its causes and led to the development of new diagnostic protocols, particularly in Brazil [42,50,52,53].

In Brazil, chromosomal abnormalities accounted for 6–8% of microcephaly cases, with a prevalence ranging from 0.18 to 0.36 per 10,000 births. Non-chromosomal syndromes contributed to 4–7% of cases, with prevalence estimates between 0.16 and 0.22 per 10,000. Across other Latin American countries, chromosomal microcephaly ranged from 0.4 to 2% (0.01–0.02 per 10,000 births), while non-chromosomal syndromes ranged from 1 to 3% (0.02–0.04 per 10,000). Although overall prevalence increased, the proportion of genetically attributed cases declined, indicating a higher detection of ZIKV-associated cases and possibly milder phenotypes due to improved diagnostic protocols [50].

In Mexico, microcephaly surveillance was conducted through RyVEMCE and the Dirección General de Epidemiología. Prior to 2015, reporting emphasized neural tube defects. The emergence of ZIKV led to mandatory craniofacial screening, and the implementation of the “Diagnosis of Microcephaly Cases” protocol to identify both ZIKV-associated and genetically induced cases. However, the genetic component has received less attention, and no specific reports on genetic etiologies have been published [46,51].

### 3.2. Reported Genetic Variants Associated with Microcephaly in Latin America

Understanding whether microcephaly arises from environmental or genetic causes is critical for designing targeted prevention and management protocols. Interventions such as folic acid fortification and the dissemination of diagnostic manuals through ECLAMC exemplify this preventive approach [42,43,50].

This section focuses on familial and autosomal recessive forms of microcephaly. In 2003, Leal et al. reported a Brazilian family practicing consanguinity, in which seven individuals exhibited a CENPJ gene mutation (c.3704T>A; p.Glu1235Val) resulting in severe microcephaly and intellectual disability [54,55]. In Argentina, Cueto-González et al. (2020) [55] described a case involving compound heterozygous truncating variants in *CENPJ* (c.289dupA; p.Thr97Asnfs7 and c.1132C>T; p.Arg378) in a patient with progressive microcephaly and severe cognitive impairment. Both parents were phenotypically normal and non-consanguineous. Variant frequencies in Latin America are estimated at 0.014% and 0.012%, respectively, based on ClinVar data.

### 3.3. Holoprosencephaly in Latin America

Holoprosencephaly (HPE), the most prevalent structural brain anomaly during embryogenesis, occurs in 1 in 250 pregnancies and in 0.6–0.8 per 10,000 live births. The clinical spectrum includes midline facial defects, microtia, cleft lip/palate, and skeletal abnormalities, often prompting more robust case reporting than isolated microcephaly [49,56].

Several monogenic causes of HPE have been reported in Latin America. In 2007, El-Jaick et al. identified *TGIF1* variants (including p.Glu45*, p.Ser46fs, and p.His76Gln) in patients from the U.S. and Brazil. Notably, one case involved a frameshift mutation transmitted from a father with only mild features (hypotelorism, anosmia) [57]. In 2008, Costa-Lima et al. described a *ZIC2* duplication (c.718_720dupCAC), not typically associated with HPE, in a fetus with anencephaly whose father was homozygous for the variant [58]. Aguinaga et al. (2011) reported a *SHH* nonsense variant (c.384G>A; p.Trp127*) in a Mexican man with a history of recurrent HPE miscarriages [59]. Other familial cases include a Brazilian family with a *ZIC2* polyalanine expansion (p.Val471Argfs*57), resulting in semilobar HPE and microcephaly in two children [60].

According to ECLAMC data, Chile’s HPE prevalence was 0.69 per 10,000 births from 1972 to 2012, with 12.7% of cases presenting with microcephaly and another 12.7% with chromosomal abnormalities (including trisomy 13 and 18) [49].

De Castro et al. (2021) [61] reported 27 HPE cases from Argentina, Brazil, and Peru, with 51.9% exhibiting microcephaly and 40.7% carrying pathogenic variants in *SHH*, *SIX3*, *ZIC2*, and *TGIF1*. Most of these genetic variants were de novo and correlated with more severe phenotypes, although the presence of multiple variants did not always predict severity. Environmental factors during gestation likely modulate the clinical outcome.

The prognosis for HPE with microcephaly is generally poor, particularly when multiple neurodevelopmental genes are affected. Penetrance varies by gene: *TGIF1* (10%), *SHH* (~50%), and *SIX3* (complete in hemizygous state) [57,58,61,62].

### 3.4. Diagnostic and Medical Approach to Microcephaly Cases

Since 2009, the American Academy of Neurology (AAN) has established clinical guidelines for the evaluation and management of microcephaly, which have been adopted by congenital malformation surveillance systems such as ECLAMC and the National Birth Defects Registry (DAN) in Mexico [50,51,63,64]. The diagnostic process begins with a comprehensive review of the patient’s medical history, including familial instances of microcephaly or parental consanguinity. The maternal clinical history is also evaluated, with particular attention to chronic or metabolic conditions, exposure to teratogenic agents (e.g., congenital infections), intrauterine growth restriction, and maternal malnutrition.

The next step involves a detailed physical examination. Measurements of weight, length, and head circumference are compared to the INTERGROWTH-21st standards recommended by the World Health Organization (WHO). Upon confirming microcephaly, it is essential to determine whether it is congenital or acquired postnatally, syndromic or non-syndromic, static or progressive, associated with neurological deficits, or represents a case of relative microcephaly.

If an etiology can be suspected based on clinical and historical data, targeted diagnostic testing—such as screening for infectious agents, metabolic disorders, or genetic abnormalities—is warranted. In cases where no etiology is immediately identifiable, neuroimaging is recommended at the time of diagnosis, and again at approximately two years of age, when myelination is complete and structural brain abnormalities are more readily detected [50,51,63,65,66].

All patients should undergo neurological evaluation and benefit from a multidisciplinary approach, especially those presenting with neurological impairment. Treatment options are determined by the underlying cause. For example, in metabolic conditions such as phenylketonuria, early dietary management can mitigate neurological complications. In cases of microcephaly related to maternal malnutrition, postnatal nutritional rehabilitation may support improved neurodevelopmental outcomes. For microcephaly of genetic origin, genetic counseling is advised to inform families about the natural history of the disorder, recurrence risks, potential interventions, and to offer psychosocial support as needed.

Children with neurodevelopmental disorders should receive early intervention services, including physical, occupational, and speech therapies. Pharmacological treatment is recommended for seizure control to prevent further neurological sequelae [63,65,66].

ECLAMC advocates for primary prevention through comprehensive prenatal care, including regular check-ups, proper maternal nutrition, and supplementation with folic acid and vitamin B12 during pregnancy. Monitoring and management of maternal chronic conditions and minimizing exposure to teratogenic agents, particularly infectious pathogens, are also emphasized. Prenatal diagnosis of congenital anomalies is considered a form of secondary prevention, as it depends on identifying the cause to enable timely surveillance and intervention, which ultimately supports primary prevention strategies [50].

## 4. Discussion

It has been mentioned previously that the systems for monitoring congenital abnormalities in Latin America (ECLAMC, ReLAMC, and ICBDSR) are interconnected and provide mutual feedback. However, there are flaws in the recording of cases due to the methodology used [43]. With respect to ECLAMC, and ReLAMC in Latin America, and RyVEMCE in Mexico, the report is voluntary by the hospital sites that are associated with the surveillance systems [46,67]. When a case of microcephaly is reported, both the ECLAMC and the Mexican surveillance system typically investigate a genetic etiology only after infectious or environmental causes have been ruled out. In contrast, the ReLAMC network and the DAN system in Mexico mandate the collection of biological samples in all cases suspected of congenital infection, particularly those potentially associated with Zika virus (ZIKV) [51,53,68].

One of the deficiencies of the hospital reporting system is that only cases of congenital abnormalities are recorded in the hospital of birth, according to the examinations and physical examination by the doctor of first contact. There is a time limit for reporting cases according to the system (ECLAMC between 12 and 24 h, in Mexico, the first 24 h). Due to this protocol, cases of secondary microcephaly are not detected, and thus no incidence report of such a case is issued, and its etiology is not investigated [49,50,67,69].

In Mexico, the RyVEMCE only covers 3. 5% of annual births; therefore, from 2013 onwards, the registration of birth certificates and fetal death certificates has become compulsory for the construction of national statistics by the National Institute of Statistics and Geography (INEGI), and with this also the reporting of genetic abnormalities in the National Health Information System (INAIS). The main disadvantage is that there is no national system to standardize both reports, as well as the inter-hospital network that feeds the ReLAMC in Nuevo León, which causes an overestimation of cases [44].

In other epidemiological reporting systems considered within the ICBDSR, case monitoring is multidisciplinary, i.e., hospital, demographic reporting, and for longer periods of time. In the particular case of Australia, they have case records up to 15 years after birth, so hospitals that follow up the patient can continue to issue reports [67]. So, a key opportunity for strengthening microcephaly surveillance in Latin America lies in expanding case detection beyond the immediate postnatal period. Future strategies should incorporate routine pediatric growth monitoring and integrate surveillance into national demographic and health surveys. This broader approach would enable the identification of late-onset or previously undiagnosed cases, thereby improving the accuracy of prevalence estimates. In Mexico, ongoing efforts to standardize national surveillance protocols aim to reduce inconsistencies and mitigate the risk of overestimating case numbers resulting from heterogeneous diagnostic and reporting practices. Harmonizing these methodologies across regions will be critical to achieving reliable, comparable data for public health planning and research.

Although, before the ZIKV epidemic, the reporting of patients with microcephaly focused on severe cases, new protocols are now in place for the reporting of cases with a medium phenotype, with an increased incidence even being detected in regions where the Arbovirus transmitting vector is not found ecologically. One example was Chile, which saw an increase in the prevalence of microcephaly during and after the pandemic, highlighting the improvement of its epidemiological reporting system, mainly in making the physical examination of first-contact doctors more strenuous [43,49,51,52].

In the case of Mexico, the search for cases of birth defects of infectious etiology is of the utmost importance, as these cases may be preventable, such as cytomegalovirus, syphilis, and congenital rubella, all of which cause microcephaly. From 2016, in the guide for reporting birth defects (DAN), protocols were implemented for the search for microcephaly related to Zika Congenital Syndrome (ZCS) and genetic causes at a national level through the program “Determination of microcephaly”, indicating clinical and ecological characteristics to consider in the case studies. The protocol’s deficiency is the loss of traceability of cases of microcephaly at birth that are not associated with ZIKV, since the request for the study depends on the clinical interpretation and expertise of the attending physician, as well as the consent of the patient’s guardians, in addition to the fact that the search for genetic factors is often interrupted when there is already a diagnosis of an infectious cause [46,51,52].

### 4.1. Study Limitations

This study faces several significant limitations that constrain the generalizability and depth of its findings. First, access to comprehensive and standardized data remains restricted. For instance, the ECLAMC database is only available to participating hospitals and institutions, which limits external validation and cross-institutional analysis. In the Mexican context, while cases of microcephaly and holoprosencephaly have been reported since 2016, etiological categorization is often restricted to infectious, environmental, or maternal comorbidity factors. Genetic etiologies are largely underreported, and although some cases reference family history, these mentions are not systematically explored.

Second, there is a notable absence of large-scale population-based association studies in Latin America. Existing genetic insights are mostly derived from isolated familial case series, such as a well-documented Brazilian family or individual cases investigated due to recurrent pregnancy loss. The high cost of molecular and cytogenetic testing contributes to this gap, along with clinical practices that often exclude mild microcephaly cases from further genetic evaluation if no neurological impairment is observed.

Third, while some progress has been made in understanding holoprosencephaly (HPE)—particularly through the identification of variants in genes such as *SHH*, *SIX3*, *ZIC2*, and *TGIF1*—most of these findings originate from non-Latin American cohorts. The focus continues to be on case-by-case or family-based studies rather than broader population-level analyses.

Additionally, there is a reliance on clinical inference rather than molecular confirmation in determining etiology. The national Mexican guidelines for congenital anomalies (DAN) recommend MLPA as an alternative to karyotyping, but only after infectious and environmental causes have been ruled out. Notably, these guidelines emphasize ruling out congenital Zika virus syndrome (CZS) and other non-genetic causes first, potentially leading to underdiagnosis of genetic contributions. Furthermore, not all MLPA kits offer robust internal validation. For example, SALSA kits P070 and P036 rely heavily on reproducibility and comparison with previously confirmed normal karyotypes, which may limit diagnostic accuracy.

From a broader perspective, several systemic limitations also affect the current understanding. There is a lack of publicly accessible, population-wide datasets in Latin America. Many publications are based on hospital-specific reports, and monogenic factors are often excluded unless studies are explicitly designed to investigate them. The literature may also reflect publication bias, with a tendency to report severe phenotypes, although moderate phenotypes were historically underreported, especially before the ZIKV epidemic. Diagnostic thresholds also vary: Brazil, for example, initially included only cases with head circumference below three SDs, potentially excluding milder cases.

Lastly, findings from high-income countries may not be directly extrapolatable to Latin American contexts due to differences in healthcare infrastructure, economic resources, and data reporting mechanisms. In Mexico, for example, although national guidelines encourage online reporting of congenital anomalies, this system primarily involves institutional healthcare personnel and may overlook births attended by midwives, who are often the first point of care in low-resource regions.

### 4.2. Future Directions

To advance the understanding of genetically driven congenital anomalies such as microcephaly and holoprosencephaly in Latin America, several key areas require strategic development.

First, there is an urgent need for population-based genomic studies in the region. Current knowledge relies heavily on isolated case reports and family-based analyses, which, while informative, do not reflect the broader genetic landscape. Investment in large-scale genomic epidemiology initiatives, including biobanks and national registries, would allow for the identification of both common and rare variants associated with these conditions.

Second, systematic integration of genetic testing into clinical practice is essential. Guidelines should promote early genetic screening—including chromosomal microarray, MLPA, and next-generation sequencing—as part of routine diagnostic workflows for congenital anomalies, even in cases without overt neurological impairment. Ensuring the availability of validated and standardized diagnostic kits will be critical to improve accuracy and reproducibility.

Third, surveillance systems must evolve to capture a wider range of phenotypic presentations and etiological data. This includes improving reporting platforms to incorporate genetic findings, standardizing definitions and diagnostic criteria, and ensuring mandatory reporting from both public and private healthcare sectors. Moreover, midwives and other frontline birth attendants should be included in the surveillance ecosystem, particularly in underserved areas where they serve as the first and often only point of contact.

Fourth, capacity building and funding mechanisms must be established to support genetic research and diagnostics in low- and middle-income settings. This involves training healthcare professionals in clinical genetics, expanding laboratory infrastructure, and facilitating international collaborations to bridge technological and expertise gaps.

Finally, addressing publication and ascertainment biases is critical. Future research should aim to include mild and atypical cases, and avoid the overrepresentation of severe phenotypes that may distort our understanding of prevalence and spectrum. Similarly, greater emphasis should be placed on context-specific data generation rather than extrapolating findings from high-income countries, ensuring that local genetic diversity and environmental exposures are adequately captured in research designs.

In summary, a coordinated effort to expand genetic surveillance, diagnostic capacity, and research equity will be vital to uncover the true burden and etiology of congenital anomalies in Latin America, and, ultimately, to inform more inclusive and effective public health interventions.

## 5. Conclusions

Although Latin America has maintained an epidemiological surveillance system for congenital anomalies since 1967, the detection and reporting of microcephaly only gained greater prominence following the Zika virus (ZIKV) epidemic. Prior to this, epidemiological efforts tended to focus on congenital anomalies presenting with more overt and severe phenotypes, often neglecting microcephaly, which can manifest with more subtle cranial measurements that may not meet the diagnostic thresholds established by international standards. This discrepancy increases the risk of underdiagnosis and underreporting of microcephaly cases in the region.

In Mexico, current surveillance strategies for microcephaly primarily focus on identifying infectious and environmental causes, in alignment with public health priorities aimed at preventing teratogenic exposures. Moving forward, it is essential to integrate systematic genetic investigations into surveillance protocols, particularly for cases presenting with severe or syndromic phenotypes. Expanding the etiological assessment to include genomic analyses will not only enhance diagnostic precision but also contribute to a more comprehensive understanding of the underlying causes of microcephaly. This integrated approach can support more personalized medical management and inform genetic counseling, while also uncovering previously unrecognized patterns of inheritance or gene–environment interactions.

### Methodology

A structured literature search was conducted in PubMed and Google Scholar using MeSH terms and keywords related to the genetic causes of microcephaly reported in Latin America. The search terms included: “microcephaly”, “anencephaly”, “reduced cephalic perimeter”, “surveillance guidelines in Latin America”, “ECLAMC”, “ReLAMC”, “ICBDSR”, “RyVECEM”, “genetic causes of microcephaly”, “syndromic microcephaly”, “non-syndromic microcephaly”, “monogenic microcephaly”, and “congenital abnormalities in Latin America”.

Inclusion criteria encompassed original research articles, narrative reviews, and systematic reviews that focused on microcephaly in Latin America with a confirmed or suspected genetic etiology. The selected studies were categorized into two groups: (1) those describing the molecular basis and pathophysiology of genetically derived microcephaly, and (2) those focusing on the evolution of surveillance systems for congenital anomalies in Latin America, both before and after the Zika virus (ZIKV) outbreak.

## Figures and Tables

**Table 1:** Alterations associated with microcephaly.

	Name of the Disease	Genetic Alterations	Percentage of Cases with Microcephaly	Relevant Clinical Features	Source
** Structural and numerical abnormalities **	Patau’s Syndrome	Mosaicism, complete or partial trisomy of chromosome 13	86% complete trisomy 52% mosaicism 29% 13q partial trisomies	Incomplete midline cleavage, leading to holoprosencephaly in varying degrees.	[7,8,9]
	Down Syndrome	Mosaicism or complete trisomy of chromosome 21	ND	Neurological and metabolic disorders, dysmorphic facial features with 100% penetrance.	[10,11]
	Edwards Syndrome	Mosaicism, complete or partial trisomy of chromosome 18	8–100%	Microcephaly, microphthalmia, microretrognathia, skeletal and cardiac defects.	[12,13]
	Cri-Du-Chat Syndrome	Deletion on the short arm of chromosome 5	91.1%	Dysmorphic facial features, developmental and growth delays, patients with a distinctive “cat-like cry”.	[11,12]
	Wolf–Hirschhorn Syndrome	Deletion on chromosome 4p16	~90%	Craneofacial appearance like “Greek soldier’s helmet”, microcephaly, micrognathia, and convulsive episodes in 93%.	[14,15,16,17]
** Monogenic syndromes **	Smith–Lemil–Opitz Syndrome	Deficiency 7-dehydrocholeterol reductase (*7-DHC*) enzyme	80–84%	Microcephaly, syndactyly in toes, and polydactyly in hands, cleft lip and palate, intestinal motility failure, genital abnormalities in 70%, medium to severe intellectual disability.	[15,18,19]
	Feingold Syndrome 1 and 2	Pathogenic variant of *MYCN and MIR17HG* genes, respectively	86%	Hand and facial abnormalities, clinodactyly in the second and fifth fingers; hypoplasia of the middle phalanx, dysmorphic facial features, tracheo-esophageal fistula, intellectual disability. Penetrance in 100% in digital abnormalities.	[15,20]
	Cornelia de Lange Syndrome	Heterozygous mutation in *NIPBL* (80%), *SMC1A* (5%), *HDAC8* (4%), *SMC3* (1–2%), *RAD21* (<1%), *BRD4* (<1%) genes	>90%	Dysmorphic facial features, prominent and arched eyebrows, growth restriction, oligodactyly, reduction in the size of the upper extremities, intellectual disability ranging from mild to severe.	[21,22]
	Miller–Dieker Syndrome	Deletion of the 17p13 region (involvement of *PAFAH1B1* and *YWHAE* genes).	ND	Lissencephaly, congenital or acquired microcephaly during the first year of life, micrognathia, cardiac renal and ocular malformations.	[15,23,24]
	Seckel Syndrome	Genetic mutations of *ATR*, *CENPJ*, *CEP152*, *CEP63*, *DNA2*, *NIN*, *NSMCE2*, *RBBP8*, *TRAIP* genes	ND	Short stature, microcephaly, intellectual disability, and craneofacial features described as “bird-headed”.	[25,26,27]

ND: Not described.

## Data Availability

No new data were created or analyzed in this study. Data sharing is not applicable to this article.

## Abbreviations

The following abbreviations are used in this manuscript:

MCPH	Autosomal Recessive Primary Microcephaly
HPE	Holoprosencephaly
ECLAMC	Latin American Collaborative Study of Congenital Malformations
RyVEMCE	the Registry and Epidemiological Surveillance of External Congenital Malformations
ZACS	Zika Associated Congenital Syndrome
ICBDSR	International Clearinghouse for Birth Defects Surveillance and Research
